# Identification and Characterization of a Novel, Cold-Adapted d-Xylobiose- and d-Xylose-Releasing Endo-β-1,4-Xylanase from an Antarctic Soil Bacterium, *Duganella* sp. PAMC 27433

**DOI:** 10.3390/biom11050680

**Published:** 2021-04-30

**Authors:** Do Young Kim, Jonghoon Kim, Yung Mi Lee, Jong Suk Lee, Dong-Ha Shin, Bon-Hwan Ku, Kwang-Hee Son, Ho-Yong Park

**Affiliations:** 1 Industrial Bio-Materials Research Center, KRIBB, Daejeon 34141, Korea; kdy119@kribb.re.kr (D.Y.K.); kjh1018@kribb.re.kr (J.K.); sonkh@kribb.re.kr (K.-H.S.); 2 Division of Life Sciences, Korea Polar Research Institute, Incheon 21990, Korea; ymlee@kopri.re.kr; 3 Biocenter, Gyeonggido Business & Science Accelerator (GBSA), Suwon 16229, Korea; leejs@gbsa.or.kr; 4 Insect Biotech Co. Ltd., Daejeon 34054, Korea; dhshin@insectbiotech.co.kr (D.-H.S.); balmania@insectbiotech.co.kr (B.-H.K.)

**Keywords:** Antarctic bacterium, *Duganella* sp., GH10, cold-adapted, endo-β-1,4-xylanase

## Abstract

Endo-β-1,4-xylanase is a key enzyme in the degradation of β-1,4-d-xylan polysaccharides through hydrolysis. A glycoside hydrolase family 10 (GH10) endo-β-1,4-xylanase (XylR) from *Duganella* sp. PAMC 27433, an Antarctic soil bacterium, was identified and functionally characterized. The XylR gene (1122-bp) encoded an acidic protein containing a single catalytic GH10 domain that was 86% identical to that of an uncultured bacterium BLR13 endo-β-1,4-xylanase (ACN58881). The recombinant enzyme (rXylR: 42.0 kDa) showed the highest beechwood xylan-degrading activity at pH 5.5 and 40 °C, and displayed 12% of its maximum activity even at 4 °C. rXylR was not only almost completely inhibited by 5 mM *N*-bromosuccinimide or metal ions (each 1 mM) including Hg^2+^, Ca^2+^, or Cu^2+^ but also significantly suppressed by 1 mM Ni^2+^, Zn^2+^, or Fe^2+^. However, its enzyme activity was upregulated (>1.4-fold) in the presence of 0.5% Triton X-100 or Tween 80. The specific activities of rXylR toward beechwood xylan, birchwood xylan, oat spelts xylan, and *p*-nitrophenyl-β-d-cellobioside were 274.7, 103.2, 35.6, and 365.1 U/mg, respectively. Enzymatic hydrolysis of birchwood xylan and d-xylooligosaccharides yielded d-xylose and d-xylobiose as the end products. The results of the present study suggest that rXylR is a novel cold-adapted d-xylobiose- and d-xylose-releasing endo-β-1,4-xylanase.

## 1. Introduction

β-1,4-d-xylan is a primary hemicellulosic polysaccharide in hardwood trees. It is constituted of d-xylose molecules linked by β-1,4-d-xylosidic bonds in the backbone of the polysaccharide chains. These d-xylose-based hemicelluloses are often found in a specific form with different side-chain substitutions such as either acetyl, glucuronopyranosyl, α-l-arabinofuranosyl, 4-*O*-methyl-d-glucuronopyranosyl, feruloyl, *p*-coumaroyl groups, or in combination [1]. In ecosystems, the biological recycling of such polysaccharides is primarily performed by various microorganisms including bacteria, yeasts, and filamentous fungi, which produce exo- and endo-type β-1,4-d-xylan-degrading glycoside hydrolases (GHs) [2].

Biocatalysts including xylanolytic GH enzymes are an attractive option for sustainable and clean manufacturing technologies owing to their numerous advantages; they are biodegradable, non-toxic, have low energy consumption, and outstanding catalytic properties. Endo-β-1,4-xylanases (EC 3.2.1.8) are the key enzymes responsible for the deconstruction of β-1,4-d-xylosic materials [3] and thus, have drawn much attention as promising biocatalysts for green chemistry in the bio-based industries [4]. Based on their amino acid sequence similarities, microbial endo-β-1,4-xylanases are currently categorized into eight GH families (5, 6, 8, 10, 11, 30, 43, and 141) (http://www.cazy.org/Glycoside-Hydrolases.html) [5]. The majority of these biocatalysts are distributed between GH families 10 and 11 [6]. Retaining GH10 endo-β-1,4-xylanases possess a (β/α)_8_-barrel as a catalytic domain, while retaining GH11 endo-β-1,4-xylanases have a β-jelly roll as a catalytic domain [7].

So far, a wide variety of β-1,4-d-xylan-degrading bacterial and fungal species have been isolated from natural environments such as soil, compost, fresh water and seawater, sediment, hot springs, the digestive tracts of animals and invertebrates, etc [1,6,8,9]. Accordingly, like other microbial hemicellulases [2], many mesophilic and thermophilic endo-β-1,4-xylanases with different molecular structures and substrate specificities have also been identified and characterized [4,10]. However, only four cold-adapted or cold-active endo-β-1,4-xylanases from bacterial and fungal species in Antarctica have been genetically and functionally characterized to date [11,12,13,14], although some such enzymes from non-Antarctic organisms have been characterized at a molecular level [15,16,17,18]. In particular, 19 xylanolytic filamentous fungi were recently isolated from marine and terrestrial Antarctic environments [19] but the molecular properties of their related enzymes have not been characterized to date. In the biotechnological context, cold-adapted endo-β-1,4-xylanases can fill an important and unique gap as biocatalysts in low-temperature processes such as bioremediation of cold environments, and food and textile processes that are carried out at low temperatures [1,20]. Therefore, to explore such new enzymes with distinct molecular and biocatalytic characteristics, we isolated some psychrotolerant β-1,4-d-xylan-decomposing bacteria from a soil sample from King George Island, Antarctica (62°13′50.8′′ S; 58°46′30.9′′ W). Herein, we describe the genetic and functional characteristics of a novel cold-adapted GH10 endo-β-1,4-xylanase (XylR) identified from the whole genome sequence of *Duganella* sp. PAMC 27433.

## 2. Materials and Methods

### 2.1. Chemicals

A series of d-xylooligosaccharides of d-xylobiose (X_2_) to d-xylohexaose (X_6_), wheat arabinoxylan, ivory nut mannan, and curdlan from *Alcaligenes faecalis* were provided by Megazyme International Ireland Ltd. (Wicklow, Ireland). Chitosan was obtained from USB Co. (Cleveland, OH, USA). All other chemical compounds containing d-xylose, *p*-nitrophenyl (*p*NP)-sugar derivatives, Avicel PH-101, birchwood xylan, beechwood xylan, oat spelts xylan, lignin, locust bean gum, carboxymethylcellulose, and chitin from crab were purchased from Sigma-Aldrich (St. Louis, MO, USA).

### 2.2. Isolation and Identification of a Xylanolytic Microorganism

To isolate xylanolytic Antarctic microorganisms, a soil sample was collected from King George Island, Antarctica (62°13′50.8′’ S; 58°46′30.9′’ W). For cultivation of bacterial isolates, 0.5 g of the soil sample was serially diluted up to 10^−2^ using sterile 0.85% NaCl, after which 100 μL aliquots of the diluted sample suspensions were spread on 0.01× nutrient agar medium and incubated at 20 °C for 14 d. Of the heterotrophic bacteria grown on the solid medium, strain PAMC 27433 was purely isolated by culturing it on Reasoner’s 2A (R2A) agar medium three or more times. Extraction of genomic DNA from the isolate was performed by using the Mini Tissue DNA kit (Cosmo Genetech Co. Ltd., Seoul, Korea) in accordance with the manufacturer’s instructions. The 16S rRNA gene of strain PAMC 27433 was amplified with two universal primers (27F and 1492R), after which the resulting PCR (polymerase chain reaction) products were purified using LaboPass PCR purification kit (Cosmo Genetech Co. Ltd., Seoul, Korea) and sequenced with the same primers used for amplification. The sequence of its 16S rRNA gene was compared with those of type strains available in the EzBioCloud database (ChunLab Inc., Seoul, Korea) to find closely related species.

### 2.3. Molecular Cloning of the Endo-β-1,4-Xylanase (XylR) Gene

PCR amplification of the gene coding for mature recombinant XylR proteins was performed employing the two gene-specific oligonucleotides XylR-F (5′-CATATGGCGGAAGACACGCCGGAACC-3′) and XylR-R (5′-AAGCTTTCACGCCCGCGGCGG-3′). The designed upstream (XylR-F) and downstream (XylR-R) primers contained an NdeI restriction site and a HindIII restriction site, respectively. The genomic DNA of *Duganella* sp. PAMC 27433, which was extracted using a NucleoSpin gDNA Clean-up (Macherey-Nagel), was used as a template and the reaction was carried out using a PCR thermal cycler (TaKaRa), as previously described [21]. Briefly, the PCR mixture (50 μL) contained 2.5 units of FastStart Taq DNA polymerase (Roche), 2.5 mM of each dNTP, 100 pmol of each primer, 20 ng of template DNA, and a PCR buffer. The initial template denaturation was conducted for 4 min at 95 °C, followed by 35 cycles of 30 s at 95 °C, 30 s at 60.5 °C, and 1 min 10 s at 72 °C. After separating the amplified PCR products by electrophoresis on a 1.2% agarose gel, the desired gene products were isolated using a NucleoSpin Gel and PCR Clean-up (Macherey-Nagel, Düren, Germany). The purified gene products (1065-bp) were then inserted into a pGEM-T easy vector (Promega, Madison, WI, USA). The pGEM-T easy/*xylR* vectors, which were transformed into *Escherichia coli* DH5α competent cells, were isolated from the recombinant cells and subsequently cleaved with restriction endonucleases NdeI and HindIII to yield *xylR* fragments with the corresponding cohesive ends. After purifying the *xylR* fragments, they were cloned into a pET-28a(+) vector (Novagen, Darmstadt, Germany) with the same cohesive ends and then the constructed pET-28a(+)/*xylR* vectors were introduced into *E. coli* BL21 competent cells.

### 2.4. Overproduction and Purification of Recombinant Proteins

Overproduction of the N-terminal (His)_6_-tagged XylR (rXylR) was performed by culturing the recombinant *E. coli* BL21 cells harboring pET-28a(+)/*xylR* in a 5-L baffled flask, which included 1 L of Luria-Bertani broth (Difco) plus 25 mg/L of kanamycin, in a rotary shaker (150 rpm) for 10 h at 30 °C. The induction of the XylR gene was performed by adding 1 mM isopropyl β-d-1-thiogalactopyranoside (IPTG) after the absorbance of the culture at 600 nm reached 0.4–0.5. Following cultivation, the rXylR-expressing cells were harvested from the culture broth by centrifugation (7000× *g*) for 15 min at 4 °C and then stored at −20 °C for 3 h. To purify rXylR, the frozen cells were completely resuspended in a binding buffer [22] and disrupted by sonication. In this study, rXylR proteins were only produced in an active form and thus, the soluble fraction exhibiting high endo-β-1,4-xylanase activity toward beechwood xylan was selectively recovered by centrifugation (12,000× *g*) for 20 min at 4 °C. The active rXylR proteins were purified by affinity column chromatography employing a HisTrap HP (GE Healthcare, Uppsala, Sweden) (5 mL) column connected to fast protein liquid chromatography system (Amersham Pharmacia Biotech, Uppsala, Sweden). Elution of the recombinant enzymes from the column was done using a linear gradient of 20–500 mM imidazole at a flow rate of 2 mL/min. The active fractions containing rXylR were selectively combined and then desalted with a HiPrep 26/10 desalting column (GE Healthcare, Uppsala, Sweden) employing 50 mM sodium phosphate buffer (pH 6.0) as the mobile phase. The desalted fractions including purified rXylR proteins were collected, pooled, and used for further analysis.

### 2.5. Analysis of Proteins

Sodium dodecyl sulfate-polyacrylamide gel electrophoresis (SDS-PAGE) analysis of the denatured rXylR proteins in a 12.0% gel was conducted to determine its relative molecular mass. After completion of the electrophoresis, visualization of the proteins separated by SDS-PAGE was performed by staining the gel with Coomassie Brilliant Blue R-250. Quantitative analysis of the protein concentrations was performed by Bradford assay employing bovine serum albumin as a standard [21].

### 2.6. Enzyme Assays

Endo-β-1,4-xylanase activity was assayed by quantitatively measuring the amount of d-xylose and its reducing oligosaccharides produced from biocatalytic degradation of beechwood xylan employing 3,5-dinitrosalicylic acid (DNS) reagent and d-xylose as a standard. The standard assay mixture (0.5 mL) contained 1.0% beechwood xylan or 5 mM *p*NP-sugar derivatives together with an appropriately diluted enzyme preparation (0.05 mL) in 50 mM sodium phosphate buffer (pH 5.5). The biocatalytic reaction was initiated by adding 0.05 mL of diluted enzyme solution to the reaction mixture and achieved at 40 °C for 10 min. After completion of the enzyme reaction, 0.75 mL of the DNS reagent was added to the reaction mixture, followed by boiling for 5 min to develop the red-brown color. One unit (U) of endo-β-1,4-xylanase activity toward β-1,4-d-xylans or *p*NP-sugar derivatives was defined as the amount of rXylR required to produce 1 μmol of reducing sugar or *p*NP, respectively, per min under standard assay conditions.

### 2.7. Effects of pH, Temperature, and Chemicals on the rXylR Activity

The optimum pH of rXylR was evaluated at pH values ranging from 3.5 to 10.5 at 40 °C for 10 min employing the following buffer systems (50 mM): sodium citrate (pH 3.5–5.5), sodium phosphate (pH 5.5–7.5), Tris-HCl (pH 7.5–9.0), and glycine-NaOH (pH 9.0–10.5). The pH stability of the enzyme was investigated by determining its remaining endo-β-1,4-xylanase activity after pre-incubation of 1 h at 4 °C in the aforementioned pH buffers. In this case, the enzyme reaction was initiated by adding 1.0% beechwood xylan to the reaction mixture. The effect of temperature on the endo-β-1,4-xylanase activity of rXylR was examined by reacting the enzyme with beechwood xylan at 4, 8, 18, 30, 35, 40, 45, 50, and 55 °C for 10 min in 50 mM sodium phosphate buffer (pH 5.5). To assess the thermostability of rXylR, it was first pre-incubated at the corresponding temperature for 15, 30, and 60 min, respectively, in 50 mM sodium phosphate buffer (pH 5.5), then the substrate was added to the reaction mixture to initiate the biocatalytic reaction. The residual endo-β-1,4-xylanase activity of the enzyme was assayed for 10 min at 40 °C. The effects of divalent cations (each 1 mM) and chemical substances (each 5 mM or 0.5%) on the endo-β-1,4-xylanase activity of rXylR was determined after pre-incubation of rXylR at 4 °C for 10 min in the standard assay mixture that contained the chemical of interest.

### 2.8. Analysis of the Degradation Products

Biocatalytic degradation of birchwood xylan (2 mg/0.2 mL) or d-xylooligosaccharides (X_2_–X_6_, each 1 mg/0.2 mL) was done by reacting the β-1,4-d-xylosic substrates with rXylR (1 μg) in 50 mM sodium phosphate buffer (pH 5.5) for 2 h at 35 °C, during which time rXylR was more thermostable at the temperature than at 40 °C. The biocatalytic reaction was stopped by boiling the reaction mixtures at 100 °C for 5 min and the resulting degradation products were subsequently analyzed by liquid chromatography/tandem mass spectrometry (LC-MS/MS) using d-xylose (X_1_) and d-xylooligosaccharides (X_2_-X_6_) as standards. High performance liquid chromatography (HPLC) analysis was achieved using a Finnigan Surveyor Modular HPLC system (Thermo Electron Co., Waltham, MA, USA) equipped with an Asahipak NH2P-50 2D column (5 μm, 2.0 × 150 mm, Shodex) [23]. Moreover, LC-MS was carried out by employing a Finnigan LCQ Advantage MAX ion trap mass spectrometer (Thermo Electron Co., Waltham, MA, USA) equipped with an electrospray ionization (ESI) source [23].

### 2.9. Binding Assay

The substrate-binding ability of rXylR was evaluated using diverse insoluble polymers including Avicel PH-101, oat spelts xylan, ivory nut mannan, lignin, chitin from crab, chitosan, curdlan from *A. faecalis*, and wheat arabinoxylan. Before the binding assay, the insoluble materials were first washed four times with sterile distilled water to eliminate any residual water-soluble carbohydrates, after which they were rewashed with 50 mM sodium phosphate buffer (pH 5.5). Binding experiments of rXylR to candidate polymers with a specific microstructure were performed using the assay mixtures, which included an appropriately diluted enzyme preparation (5.0 U/mL) together with an equal volume of insoluble polymer in a 1.5 mL Eppendorf tube, on ice for 2 h. The assay mixtures were vigorously stirred every 5 min during the reaction period. The supernatants containing rXylR proteins unbound to the evaluated polymers were carefully retrieved by centrifugation (12,000× *g*) and applied directly to the quantitative assay of protein concentration and endo-β-1,4-xylanase activity.

## 3. Results and Discussion

### 3.1. Identification of a Xylanolytic Antarctic Isolate

A Gram-negative bacterium, strain PAMC 27433, was identified as *Duganella* sp. based on its 16S rRNA gene sequence (GenBank accession number: MW820638) that was 98.2% similar to that of *Duganella pernnla* FT109W^T^. Strain PAMC 27433 was deposited in the Polar and Alpine Microbial Collection under code no. *Duganella* sp. PAMC 27433.

### 3.2. Molecular Characterization of the GH10 Endo-β-1,4-Xylanase Gene

**Figure 1:**
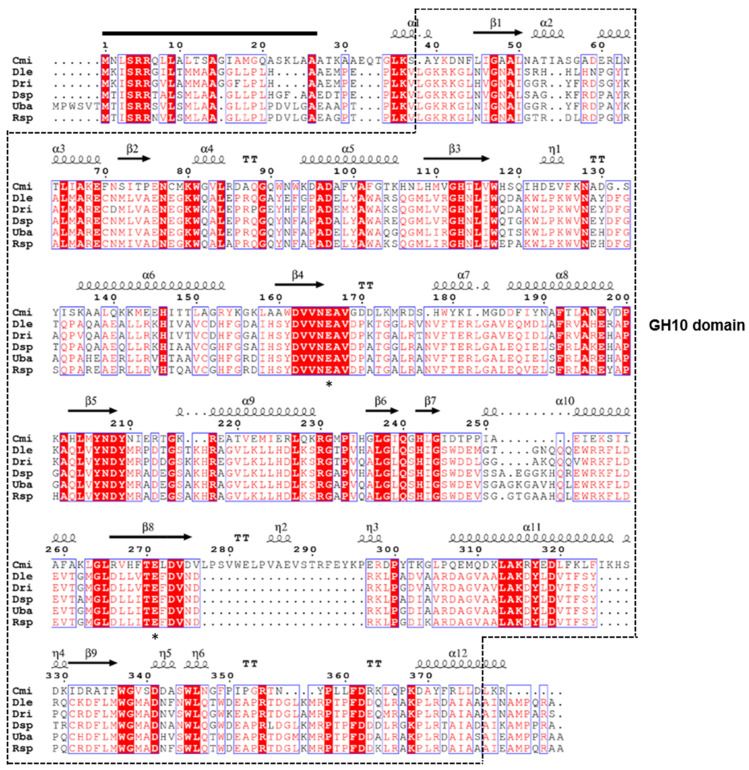
Molecular structure of *Duganella* sp. PAMC 27433 GH10 endo-β-1,4-xylanase and structure-based sequence alignment of the enzyme with its structural homologs. The first line shows the secondary structure elements (α-helix, squiggle; β-strand, arrow; 3_10_-helix, η; β-turn, TT) of *Cellvibrio mixtus* ATCC 12,120 endo-β-1,4-xylanase (PDB code: 1UQY) used as a template. Sequences (GenBank accession numbers): Cmi, *Cellvibrio mixtus* ATCC 12,120 endo-β-1,4-xylanase (AAD09439); Dle, *Duganella levis* endo-β-1,4-xylanase (WP_161054863); *Duganella rivi* β-1,4-xylanase (WP_154357092); *Duganella* sp. PAMC 27433 GH10 endo-β-1,4-xylanase (MW735678); uncultured bacterium BLR13 endo-β-1,4-xylanase A precursor (ACN58881); and *Rugamonas* sp. SG757 endo-β-1,4-xylanase (WP_176653060). The predicted signal peptide is indicated by a black bar and GH10 domain is outlined by dotted line. Highly conserved amino acid residues, which play an important role in biocatalytic reaction, are displayed by asterisks.

The XylR gene (GenBank accession number: MW735678) of the xylanolytic Antarctic bacterium *Duganella* sp. PAMC 27433 was discovered to contain a 1122-bp open reading frame that codes for an acidic protein (pI: 6.84) of 373 amino acids with a deduced molecular mass of 41,350 Da. When analyzed by the SignalP 5.0 server (http://www.cbs.dtu.dk/services/SignalP/), the premature XylR was predicted to be an extracellular protein with a signal sequence that might be post-translationally processed between Ala23 and Ala24 in the N-terminus region (Figure 1). On the other hand, the (His)_6_-tagged recombinant XylR lacking a signal peptide evaluated in this study was predicted to be a polypeptide with a calculated pI of 6.79 and a deduced molecular mass of 41,234 Da. Protein BLAST and Pfam analyses of the primary structure of XylR indicated that the enzyme might be a non-modular carbohydrolase made up of a single catalytic GH10 domain (from Leu31 to Asp364).

Sequence alignment between the primary structure of XylR and those of its structural homologs available in the NCBI database indicated that the catalytic GH10 domain of the enzyme exhibited the highest sequence identity (86%) with that of uncultured bacterium BLR13 endo-β-1,4-xylanase A precursor (GenBank accession number: ACN58881), which had not yet been structurally and functionally characterized. Moreover, the catalytic GH10 domain of XylR shared 84, 82, and 79% sequence identities with that of *Rugamonas* sp. SG757 endo-β-1,4-xylanase (WP_176653060), *Duganella levis* endo-β-1,4-xylanase (WP_161054863), and *Duganella rivi* endo-β-1,4-xylanase (WP_154357092), respectively, which were recorded in the NCBI database only. To the best of our knowledge, no report concerning the functional characteristics of microbial GH10 endo-β-1,4-xylanases available in the NCBI database, which share sequence identity of >50% with XylR, has been published to date.

**Figure 2:**
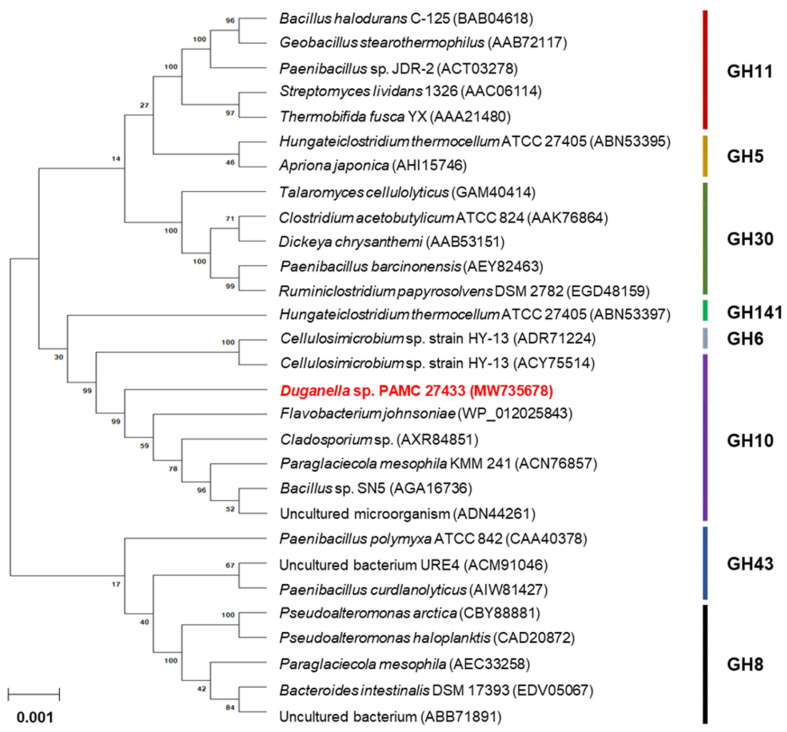
Phylogenetic analysis of *Duganella* sp. PAMC 27433 GH10 endo-β-1,4-xylanase (XylR) and its closely related functional homologs. Alignment of the amino acid sequences was carried out using ClustalW in the MegAlign program (DNASTAR Inc., Madison, WI, USA). The protein sequences employed for phylogenetic analysis were retrieved from the GenBank database.

The phylogenetic tree shown in Figure 2 also displayed that the primary structure of XylR was similar to that of the functional homologs within the family GH10, which is mainly composed of retaining endo-β-1,4-xylanase (EC 3.2.1.8), endo-β-1,3-xylanase (EC 3.2.1.32), and endo-β-1,4-glucanase (EC 3.2.1.4). The two highly conserved amino acid residues, Glu158 acting as the acid/base catalyst and Glu279 acting as the catalytic nucleophile, which may take part in the double-displacement of retaining glycoside hydrolases [24], were observed in the active site of premature XylR (Figure 1).

In this study, employing a GH10 endo-β-1,4-xylanase from *Cellvibrio mixtus* ATCC 12,120 (PDB code: 1UQY) as a template, the secondary structure elements of XylR from *Duganella* sp. PAMC 27433 were analyzed. The structure-based sequence alignment rendered using ESPript software 3.0 (https://espript.ibcp.fr/ESPript/ESPript/) exhibited that the catalytic GH10 domain in XylR was made up of 12 α-helices, 9 β-strands, 6 3_10_-helices, and 6 β-turns (Figure 1).

### 3.3. Purification and SDS-PAGE Analysis of rXylR Proteins

It has been demonstrated that when overexpressed in *E. coli* BL21, some bioactive proteins are frequently produced as insoluble inclusion bodies that are purified through an on-column protein refolding method [25]. A tri-modular GH10 endo-β-1,4-xylanase with a fibronectin type 3 domain and a carbohydrate-binding module 2 from *Cellulosimicrobium* sp. strain HY-13 [26] and a bi-modular GH10 endo-β-1,4-xylanase with a ricin-type β-trefoil lectin domain-like domain from *Luteimicrobium xylanilyticum* HY-24 [22] are examples of enzymes that were formed as inactive protein aggregates in the recombinant cells. However, similar to other non-modular GH10 endo-β-1,4-xylanases [17,18,21], rXylR comprised of only a single catalytic GH10 domain was overproduced in an active form in *E. coli* BL21. Thus, the enzyme could be simply purified to electrophoretic homogeneity by basic affinity chromatography using a HisTrap HP column.

**Figure 3:**
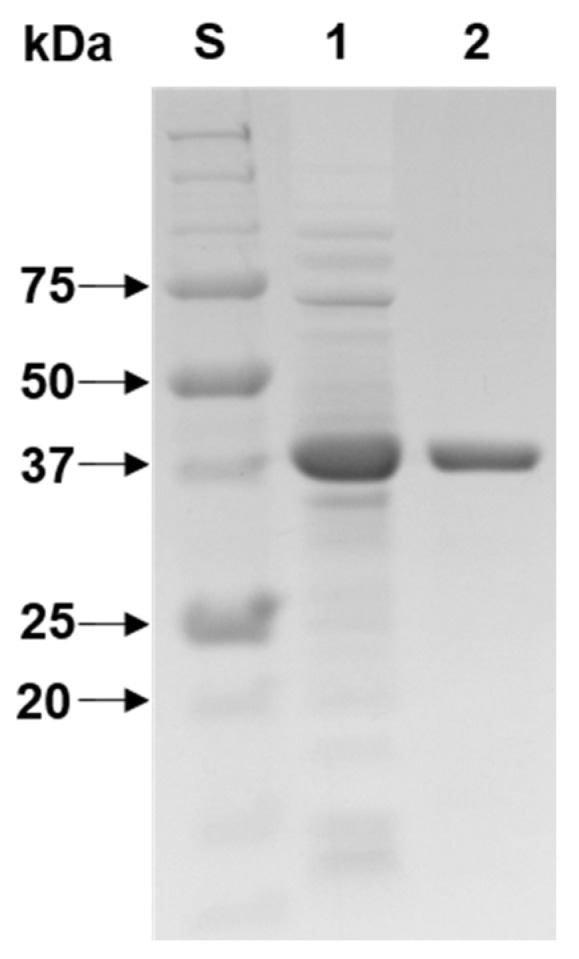
Sodium dodecyl sulfate-polyacrylamide gel electrophoresis (SDS-PAGE) of the purified rXylR after affinity chromatography on HisTrap HP. Lane S, standard marker proteins; lane 1, the soluble cell lysate of rXylR-expressing *E. coli* BL21 after IPTG induction; lane 2, purified rXylR.

As determined by SDS-PAGE (Figure 3), the relative molecular mass of purified rXylR was assessed to be 42.0 kDa and this value was in agreement with its deduced molecular mass (41,234 Da) that was analyzed by the Compute pI/MW server (https://www.expasy.org/resources/compute-pi-mw). The molecular size (42.0 kDa) of rXylR was larger than that (37.0 kDa) of a thermolabile GH10 endo-β-1,4-xylanase from the Antarctic mold *Cladosporium* sp. [13] and that (39.0 kDa) of a cold-adapted GH10 endo-β-1,4-xylanase from *Bacillus cellulosilyticus* [18], however, its molecular size was similar to that (43.0 kDa) of a cold-adapted GH10 endo-β-1,4-xylanase from the Antarctic yeast *Cryptococcus adeliae* [11] and that (43.0 kDa) of a cold-active GH10 endo-β-1,4-xylanase from the marine bacterium *Echinicola rosea* sp. nov. JL3085^T^ [27]. However, the molecular mass (42.0 kDa) of rXylR was smaller than that (48.5 kDa) of a cold-active, bi-modular GH10 endo-β-1,4-xylanase from *Flavobacterium johnsoniae* [16].

### 3.4. Biochemical Characterization of Recombinant Enzymes

**Figure 4:**
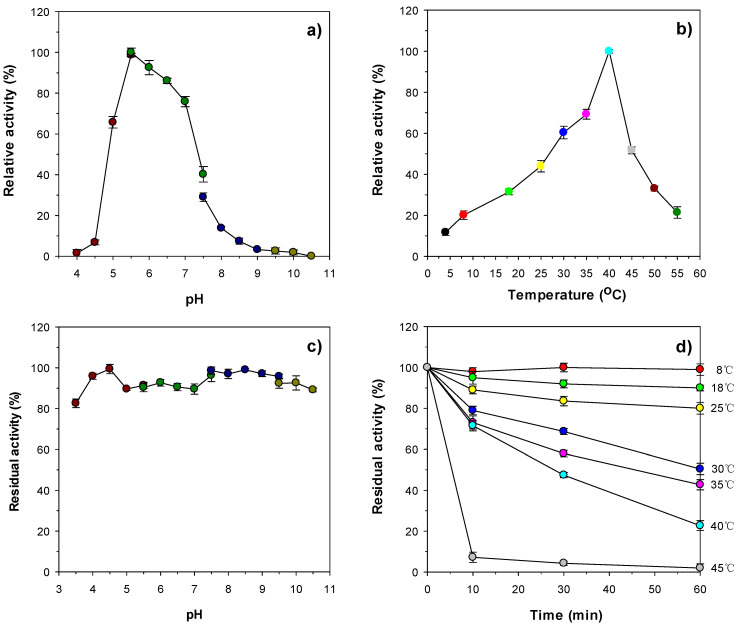
Effects of pH (**a**) and temperature (**b**) on the endo-β-1,4-xylanase activity of rXylR and effects of pH (**c**) and temperature (**d**) on the stability of rXylR. The optimal pH of rXylR was assessed using the following buffers (50 mM): sodium citrate (pH 4.0–5.5), sodium phosphate (pH 5.5–7.5), Tris-HCl (pH 7.5–9.5), and glycine-NaOH (pH 9.5–10.5). The optimum temperature of rXylM was determined at various temperatures (4–55 °C) in 50 mM sodium phosphate buffer (pH 5.5). The residual endo-β-1,4-xylanase activities were evaluated at pH 4.0–10.5 after pre-incubation of rXylR using the following buffers (50 mM) at 4 °C for 60 min: sodium citrate (pH 4.0–5.5), sodium phosphate (pH 5.5–7.5), Tris-HCl (pH 7.5–9.5), and glycine-NaOH (pH 9.5–10.5). The residual endo-β-1,4-xylanase activities were measured after pre-incubation of rXylR at 8, 18, 25, 30, 35, 40, and 45 °C in 50 mM sodium phosphate buffer (pH 5.5) for 10, 30, and 60 min, respectively. The values are mean ± SD of triplicate tests.

To date, only four endo-β-1,4-xylanases from Antarctic microorganisms have been genetically and biochemically characterized (Table 1). Of the four, two, thermolabile GH10 endo-β-1,4-xylanases from the mold *Cladosporium* sp. [13] and the yeast *C. adeliae* [28], were reported to be most active at 50 °C and pH 6.0 for beechwood xylan and at 45–50 °C in pH range 5.0–5.5 for birchwood xylan. Additionally, a GH10 endo-β-1,4-xylanase from *Psychrobacter* sp. strain 2-17 [14] and a GH8 endo-β-1,4-xylanase from *Pseudoalteromonas haloplanktis* [12] exhibited the maximum biocatalytic activity for birchwood xylan at 35 °C and pH 8.0 and at 35 °C in pH range 5.3–8.0, respectively. However, in this study, the highest endo-β-1,4-xylanase activity of rXylR for beechwood xylan was observed at 40 °C and pH 5.5 (Figure 4a,b), which suggested that the pH and temperature optima of the enzyme were distinguished from those of the aforementioned endo-β-1,4-xylanases from Antarctic microorganisms. It is interesting to note that rXylR was capable of degrading beechwood xylan even at 4 °C, although at that temperature, the enzyme showed approximately 12% of its maximum endo-β-1,4-xylanase activity. Furthermore, at 8 °C, the relative activity of rXylR to decompose the substrate was evaluated to be approximately 20% of its maximum biocatalytic activity, implying that it is a cold-adapted biocatalyst. When the enzyme reaction was performed at pH values below 5.0 or above 7.0, and at temperatures exceeding 40 °C, the endo-β-1,4-xylanase activity of rXylR was considerably reduced. However, it should be noted that rXylR was relatively stable in a broad range of pH values (3.5–10.5) similar to a cold-active GH10 endo-β-1,4-xylanase from the marine bacterium *E. rosea* sp. nov. JL3085^T^ [27]. Actually, the enzyme retained over 80% of residual endo-β-1,4-xylanase activity at those pH values even when exposed to a temperature of 4 °C for 1 h without beechwood xylan as a substrate (Figure 4c). The half-life of rXylR at 40 °C was determined to be approximately 30 min (Figure 4d), indicating that it was more stable at the temperature compared to thermolabile GH10 endo-β-1,4-xylanases from Antarctic fungal species [13,28], which were almost completely inactivated in 30 min at 40 °C. The thermostability of rXylR was gradually downregulated in a temperature-dependent manner when subjected to temperatures of 25–40 °C for the same pre-incubation period, while the enzyme was drastically inactivated when pre-incubated for 10 min at 45 °C (Figure 4d).

**Figure 5:**
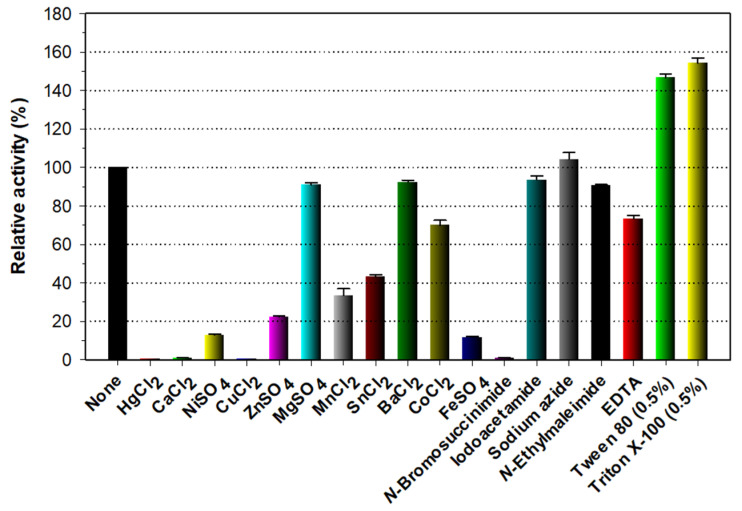
Effects of metal ions (1 mM) and chemical reagents (5 mM) on the endo-β-1,4-xylanase activity of rXylR. The values are mean ± SD of triplicate tests.

As shown in Figure 5, rXylR did not show endo-β-1,4-xylanase activity for beechwood xylan in the presence of tryptophan residue-directed modifiers such as Hg^2+^ (1 mM) and *N*-bromosuccinimide (5 mM). A similar observation was also made when some bacterial GH10 endo-β-1,4-xylanases were reacted with β-1,4-d-xylosic polysaccharides in the presence of the aforementioned compounds [5,16,22,29]. Taken together, these findings corresponded to the fact that the oxidizing substances Hg^2+^ and *N*-bromosuccinimide modify the indole ring of highly conserved tryptophan residues in the active site of GH10 endo-β-1,4-xylanases, which play a critical role in the enzyme-substrate interaction [24]. Interestingly, rXylR was not only almost completely inhibited in the presence of 1 mM Ca^2+^ or Cu^2+^ but also noticeably suppressed by 1 mM Ni^2+^, Zn^2+^, or Fe^2+^, although it was partially inhibited by 1 mM Mg^2+^, Ba^2+^, or Co^2+^. The significant inhibitory effects of the indicated divalent cations on the rXylR activity were very comparable to the inhibitory or stimulatory effects of the same compounds on the biocatalytic activity of some cold-active GH10 endo-β-1,4-xylanases [14,16,18,27,30]. For example, the metal ions (each 1 mM), Ca^2+^ and Cu^2+^, had no inhibitory effect on the activity of *Psychrobacter* sp. strain 2-17 GH10 endo-β-1,4-xylanase [14] and *Glaciecola mesophila* KMM 241 GH10 endo-β-1,4-xylanase [30]. Moreover, two GH10 endo-β-1,4-xylanases from *B. cellulosilyticus* [18] and *E. rosea* sp. nov. JL3085^T^ [27] were only slightly sensitive to 1 mM Cu^2+^, Fe^2+^, Ni^2+^, or Zn^2+^. On the other hand, in the presence of 1 mM Cu^2+^ or Fe^2+^, the biocatalytic activity of a GH10 endo-β-1,4-xylanase from *F. johnsoniae* was greatly enhanced by approximately 2.2-fold and 1.5-fold, respectively [16]. Compared to the slight suppression of *Psychrobacter* sp. strain 2-17 GH10 endo-β-1,4-xylanase by the non-ionic detergent Triton X-100 (1.0%) [14], it is noteworthy that the rXylR activity could be upregulated by >1.45-fold in the presence of 0.5% Triton X-100 or Tween 80 (Figure 5), which is similar to some GH10 functional homologs [16,23,26]. In addition, no significant inhibition or stimulation of rXylR was observed when the enzyme was pre-incubated in the presence of sulfhydryl reagents (each 5 mM) including iodoacetamide, sodium azide, and *N*-ethylmaleimide, while 1 mM ethylenediaminetetraacetic acid (EDTA) caused 27% reduction in its original endo-β-1,4-xylanase activity. The inhibitory effect of rXylR exerted by EDTA was close to that of *Psychrobacter* sp. strain 2-17 GH10 endo-β-1,4-xylanase by the same compound [14]. Conversely, the xylanolytic activity of a cold-active GH10 endo-β-1,4-xylanase from *G. mesophila* KMM 241 was slightly promoted by the metal chelator, EDTA [30].

### 3.5. Substrate Specificity and Hydrolytic Properties of rXylR

The substrate specificity of the cold-adapted rXylR enzyme, which was determined under the standard assay conditions using different types of polysaccharides and *p*NP-sugar derivatives, is listed in Table 2. Of the tested polymeric substrates, the enzyme could preferentially decompose d-xylose-based hemicelluloses with the following order: beechwood xylan > birchwood xylan > wheat arabinoxylan > oat spelts xylan. However, the biocatalytic degradation of d-glucose or d-mannose-based polysaccharides such as carboxymethylcellulose, xyloglucan, and locust bean gum by rXylR was not detectable, suggesting that it was a true β-1,4-d-xylan-specific biocatalyst lacking other glycoside hydrolase activities. The specific activity of rXylR toward beechwood xylan, birchwood xylan, wheat arabinoxylan, and oat spelts xylan was evaluated to be 274.7, 103.2, 38.9, and 35.6 U/mg, respectively. It is interesting to note that the degradation ability of rXylR toward beechwood xylan, birchwood xylan, and oat spelts xylan was notably superior to that of a cold-active GH10 endo-β-1,4-xylanase from *F. johnsoniae*, which exhibited the specific activity of 8.9, 12.0, and 9.2 U/mg, respectively, toward the same substrate polymers [16]. The beechwood xylan-degrading activity (274.7 U/mg) of the cold-adapted rXylR was also approximately 2.6- and 1.6-fold, respectively, higher than that (105 U/mg) of a cold-active GH10 endo-β-1,4-xylanase from *Bacillus* sp. SN5 [29] and that (163.8 U/mg) of a cold-adapted GH10 endo-β-1,4-xylanase from *B. cellulosilyticus* [18]. On the other hand, the β-1,4-d-xylosic polysaccharides evaluated in this study were efficiently degraded by a highly thermolabile GH10 endo-β-1,4-xylanase from the Antarctic fungus *Cladosporium* sp. in the order of wheat arabinoxylan, beechwood xylan, oat spelts xylan, and birchwood xylan with the specific activity of 670, 456, 451, and 236 U/mg, respectively [13]. When reacted with *p*NP-sugar derivatives listed in Table 2, rXylR was capable of cleaving *p*NP-β-d-cellobioside and *p*NP-β-d-xylopyranoside with a biocatalytic activity of 365.1 and 6.7 U/mg, respectively, but was not specific to *p*NP-β-d-glucopyranoside, *p*NP-β-d-mannopyranoside, and *p*NP-β-d-galactopyranoside. The hydrolysis patterns of the synthetic aryl-glycosides by rXylR were relatively similar to those of the same substrates by a mesophilic GH10 endo-β-1,4-xylanase from *Streptomyces mexicanus* HY-14 [31]. However, it has been shown that unlike rXylR, some bacterial endo-β-1,4-xylanases do not display any hydrolysis activity toward *p*NP-derivatives of d-cellobiose and d-xylose [12,22,32]. It is worth noting that the *p*NP-β-d-cellobioside-hydrolyzing activity (365.1 U/mg) of rXylR was approximately 2.1- and 2.6-fold higher, respectively, compared to that (171.7 U/mg) of *Cellulosimicrobium* sp. strain HY-13 GH10 endo-β-1,4-xylanase [26] and that (140.5 U/mg) of *Cohnella laeviribosi* HY-21 GH10 endo-β-1,4-xylanase [21]. Based on these findings, we suggest that rXylR is a novel, cold-adapted GH10 endo-β-1,4-xylanase showing distinct biocatalytic activities and substrate specificities, different from the known GH10 functional homologs.

The kinetic parameters of rXylR toward β-1,4-d-xylosic materials (each 0.2–1.5%) and *p*NP-β-d-cellobioside (1–10 mM), which were calculated by non-linear regression of the Michaelis–Menten equation, are listed in Table 3. Of the examined β-1,4-d-xylosic materials, rXylR displayed the highest *V*_max_ value of 510.9 U mg^−1^, a *K*_m_ value of 1.93 mg mL^−1^, and a *k*_cat_ value of 357.63 s^−1^ toward beechwood xylan at pH 5.5 and 40 °C. The catalytic efficiency (*k*_cat_/*K*_m_: 185.30 mg^−1^ s^−1^ mL) of rXylR for beechwood xylan was approximately 1.3- and 3.2-fold higher, respectively, than that (142.3 mg^−1^ s^−1^ mL at 40 °C) of *Bacillus* sp. SN5 GH10 endo-β-1,4-xylanase [29] and that (56.56 mg^−1^ s^−1^ mL at 30 °C) of *G. mesophila* KMM 241 GH10 endo-β-1,4-xylanase [30] for the same substrate. However, rXylR showed an apparent *k*_cat_/*K*_m_ value of 52.52, 13.63, and 13.52 mg^−1^ s^−1^ mL toward birchwood xylan, oat spelts xylan, and wheat arabinoxylan, respectively, which was substantially lower than its *k*_cat_/*K*_m_ value (185.30 mg^−1^ s^−1^ mL) toward beechwood xylan. At 40 °C, the *k*_cat_/*K*_m_ value (52.52 mg^−1^ s^−1^ mL) of rXylR for birchwood xylan was evaluated to be lower than that (64.76 mg^−1^ s^−1^ mL) of a cold-adapted GH10 endo-β-1,4-xylanase from *B. cellulosilyticus* [18] for the same polysaccharide. On the other hand, the *k*_cat_/*K*_m_ value (52.52 mg^−1^ s^−1^ mL at 40 °C) of rXylR for birchwood xylan was approximately 1.2-, 1.5-, and 24.6-fold, respectively, greater than that (42.96 mg^−1^ s^−1^ mL at 30 °C) of *G. mesophila* KMM 241 GH10 endo-β-1,4-xylanase [30], that (35.4 mg^−1^ s^−1^ mL at 25 °C) of *Psychrobacter* sp. strain 2-17 GH10 endo-β-1,4-xylanase [14], and that (2.13 mg^−1^ s^−1^ mL at 30 °C) of *F. johnsoniae* GH10 endo-β-1,4-xylanase [16] for the same substrate. It should also be noted that the *V*_max_ (649.8 U mg^−1^) and *k*_cat_/*K*_m_ (375.91 mg^−1^ s^−1^ mL) of rXylR toward *p*NP-β-d-cellobioside were approximately 1.2- and 7.1-fold, respectively, higher than its *V*_max_ (649.8 U mg^−1^) and *k*_cat_/*K*_m_ (375.91 mg^−1^ s^−1^ mL) toward beechwood xylan. In this case, compared to the *K*_m_ value (1.93 mg mL^−1^) for beechwood xylan, the lower *K*_m_ value (1.21 mM) for *p*NP-β-d-cellobioside indicated that rXylR had a higher substrate-binding affinity to *p*NP-β-d-cellobioside than beechwood xylan.

The results of HPLC analysis clearly showed that rXylR was capable of hydrolyzing birchwood xylan and d-xylooligosaccharides with a degree of polymerization in the range of 2–6, even though the susceptibility of d-xylobiose (X_2_) molecules to the enzyme was insignificant (Table 4). Enzymatic degradation of the β-1,4-d-xylosic materials resulted in the production of d-xylose (X_1_) in addition to X_2_ as the dominant end product, regardless of the degree of polymerization of the substrates. Specifically, the degradation products of birchwood xylan produced by rXylR were identified as X_1_ (37.1%) and X_2_ (62.9%). d-Xylotriose (X_3_) was also hydrolyzed by the enzyme to X_1_ (19.3%) and X_2_ (80.7%) without the formation of detectable amounts of d-xylooligosaccharides longer than X_3_, indicative of the lack of transglycosylation activity. Furthermore, the production of X_1_ in the biocatalytic reactions of d-xylooligomers and birchwood xylan was gradually increased with an increase of the β-1,4-d-xylosic substrate chain length (Table 4). Based on the hydrolytic properties of rXylR, we propose that the enzyme is the first cold-adapted X_2_ and X_1_-releasing endo-β-1,4-xylanase from Antarctic microorganisms, which can be employed as a promising candidate for the low-temperature preparation of simple sugars from β-1,4-d-xylosic materials via a one-step procedure. Considering that rXylR only produced X_1_ and X_2_ as final hydrolysis products from both d-xylooligomers (X_2_-X_6_) and birchwood xylan, the biocatalytic ability of the enzyme was very comparable to that of the known GH10 enzymes for the d-xylose-based oligomeric and polymeric substrates. However, in contrast to rXylR, a cold-active GH10 endo-β-1,4-xylanase from *G. mesophila* KMM 241 could not hydrolyze X_2_ and X_3_ [30]. In addition, the breakdown of either d-xylooligomers (X_4_–X_6_), β-1,4-d-xylans, or both, by cold-active GH10 endo-β-1,4-xylanases from *Cladosporium* sp. [13], *B. cellulosilyticus* [18], and *G. mesophila* KMM 241 [30] always accompanied the formation of either X_2_, X_3_, X_4_, or in combination, and not X_1_. Furthermore, cold-active GH10 endo-β-1,4-xylanases from *F. johnsoniae* [16] and the environmental DNA of goat rumen contents [15] were reported to produce mixtures of X_1_, X_2_, and longer d-xylooligosaccharides in the degradation processes of β-1,4-d-xylans.

### 3.6. Binding Affinity of rXylR to Hydrophobic Polymers

**Figure 6:**
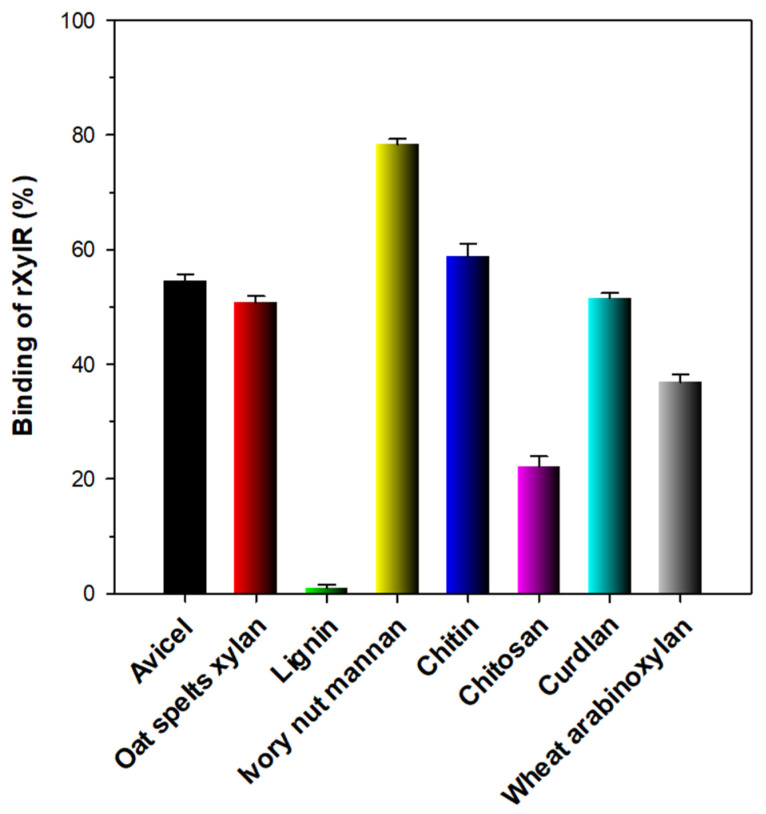
Binding of rXylR to insoluble polymers. The values are mean ± SD of triplicate tests.

In this study, the substrate-binding capacity of rXylR was investigated employing various types of hydrophobic polymers with distinct microstructures such as curdlan, Avicel PH-101, oat spelts xylan, lignin, ivory nut mannan, chitin, chitosan, curdlan, and wheat arabinoxylan (Figure 6). Unlike some modular GH10 endo-β-1,4-xylanases with a substrate-binding domain [22,26,31], the non-modular rXylR comprised of only a single catalytic GH10 domain showed relatively weak binding affinities (35–60%) to Avicel PH-101, oat spelts xylan, chitin, curdlan, or wheat arabinoxylan. Additionally, the lignin-binding capacity (<1%) of the enzyme was noticeably lower than that (>90%) of modular GH10 endo-β-1,4-xylanases from *L. xylanilyticum* HY-24 [22] and *S. mexicanus* HY-14 [31] as well as that (approx. 42%) of a non-modular GH10 endo-β-1,4-xylanase from *Microbacterium trichothecenolyticum* HY-17 [33]. On the other hand, rXylR exhibited strong binding affinity (approximately 80%) to the surface of ivory nut mannan, similar to other GH10 endo-β-1,4-xylanases [22,31,33].

## 4. Conclusions

The cold-adapted GH10 endo-β-1,4-xylanase (XylR) from an Antarctic soil bacterium, *Duganella* sp. PAMC 27433 is a novel X_2_- and X_1_-releasing biocatalyst displaying peculiar characteristics in its primary structure, sensitivity to metal ions, biocatalytic activity, substrate specificity, degradation patterns of β-1,4-d-xylosic substrates, and substrate-binding capacities to insoluble polymers. Unlike known cold-active GH10 endo-β-1,4-xylanases [16,18,30], rXylR does not have transglycosylation activity and lacks the ability to produce d-xylooligosaccharides longer than X_2_, and therefore can be exploited as a potential biocatalyst for the one-step production of fermentable sugars (X_2_ and X_1_) from various β-1,4-d-xylan polysaccharides at low temperatures. From an ecological context, the data presented in this study reflect the contribution and biological importance of xylanolytic Antarctic bacteria to the bioremediation of hemicellulosic materials in the cold environment.

## Figures and Tables

**Table 1:** Biocatalytic characteristics of cold-adapted or cold-active endo-β-1,4-xylanases from Antarctic microorganisms.

Strain	GH Family	Enzyme	M*_r_* (kDa)	Optimum pH	Optimum Temp. (°C)	*k*_cat_/*K*_m_ (mg^−1^ s^−1^ mL)	Reference
*Duganella* sp. PAMC 27433	10	rXylR	42.0	5.5	40	185.30 ^a^, 52.52 ^b^	This study
*Psychrobacter* sp. strain 2-17	10	Xyl-L	90.0	8.0	35	35.40 ^b^	[14]
*Cladosporium* sp.	10	XynA	37.0	6.0	50	NI ^c^	[13]
*Cryptococcus adeliae*	10	Xylanase	43.0	5.0–5.5	45–50	NI	[11,28]
*Pseudoalteromonas haloplanktis*	8	Xylanase	45.9	5.3–8.0	35	44.53 ^b^	[12]

^a^ Catalytic efficiency toward beechwood xylan; ^b^ Catalytic efficiency toward birchwood xylan; ^c^ Not indicated.

**Table 2:** Degradation activity of rXylR for different β-1,4-d-xylans and *p*NP-sugar derivatives.

Substrate	Specific Activity (U/mg) ^a^
Birchwood xylan	103.2 ± 2.2
Beechwood xylan	274.7 ± 1.8
Oat spelts xylan	35.6 ± 0.6
Wheat arabinoxylan	38.9 ± 1.4
Xyloglucan	ND ^b^
Locust bean gum	ND
Carboxymethylcellulose	ND
*p*NP-β-d-cellobioside	365.1 ± 2.6
*p*NP-β-d-glucopyranoside	ND
*p*NP-β-d-xylopyranoside	6.7 ± 0.2
*p*NP-β-d-mannopyranoside	ND
*p*NP-β-d-galactopyranoside	ND

The values are mean ± SD of triplicate tests; ^a^ Specific activity was obtained from the three repeated experiments; ^b^ Not detected.

**Table 3:** Kinetic parameters of rXylR determined using different β-1,4-d-xylans and *p*NP-β-d-cellobioside.

Substrate	*V*_max_ (U mg^−1^)	*K*_m_ (mg mL^−1^)	*K*_m_ (mM)	*k*_cat_ (s^−1^)	*k*_cat_/*K*_m_ (mg^−1^ s^−1^ mL)	*k*_cat_/*K*_m_ (mM^−1^ s^−1^)
Birchwood xylan	181.60	2.42		127.12	52.52	
Beechwood xylan	510.92	1.93		357.63	185.30	
Oat spelts xylan	55.51	2.85		38.85	13.63	
Wheat arabinoxylan	62.21	3.22		43.54	13.52	
*p*NP-β-d-cellobioside	649.80		1.21	454.86		375.91

Kinetic parameter values are the average of three replicates.

**Table 4:** Liquid chromatography (LC) analysis of the degradation products of β-1,4-d-xylosic materials by rXylR.

Substrate	Composition (%) ^a^ of Products Formed by Degradation	
	d-Xylose (X_1_)	d-Xylobiose (X_2_)
d-Xylobiose (X_2_)	1.8	98.2
d-Xylotriose (X_3_)	19.3	80.7
d-Xylotetraose (X_4_)	22.3	77.7
d-Xylopentaose (X_5_)	25.8	74.2
d-Xylohexaose (X_6_)	28.1	71.9
Birchwood xylan	37.1	62.9

^a^ LC area%.

## Data Availability

Not applicable.
